# Supplementary material for: Comparative Transcriptome Analysis Reveals the Innate Immune Response to Mycoplasma gallisepticum Infection in Chicken Embryos and Newly Hatched Chicks
Source: Animals (Basel). 2023 May 17;13(10):1667. doi: 10.3390/ani13101667 (PMC10215417; doi:10.3390/ani13101667)

Figure S1. The most significant enriched CCs and MFs based on FDR in chicken embryos

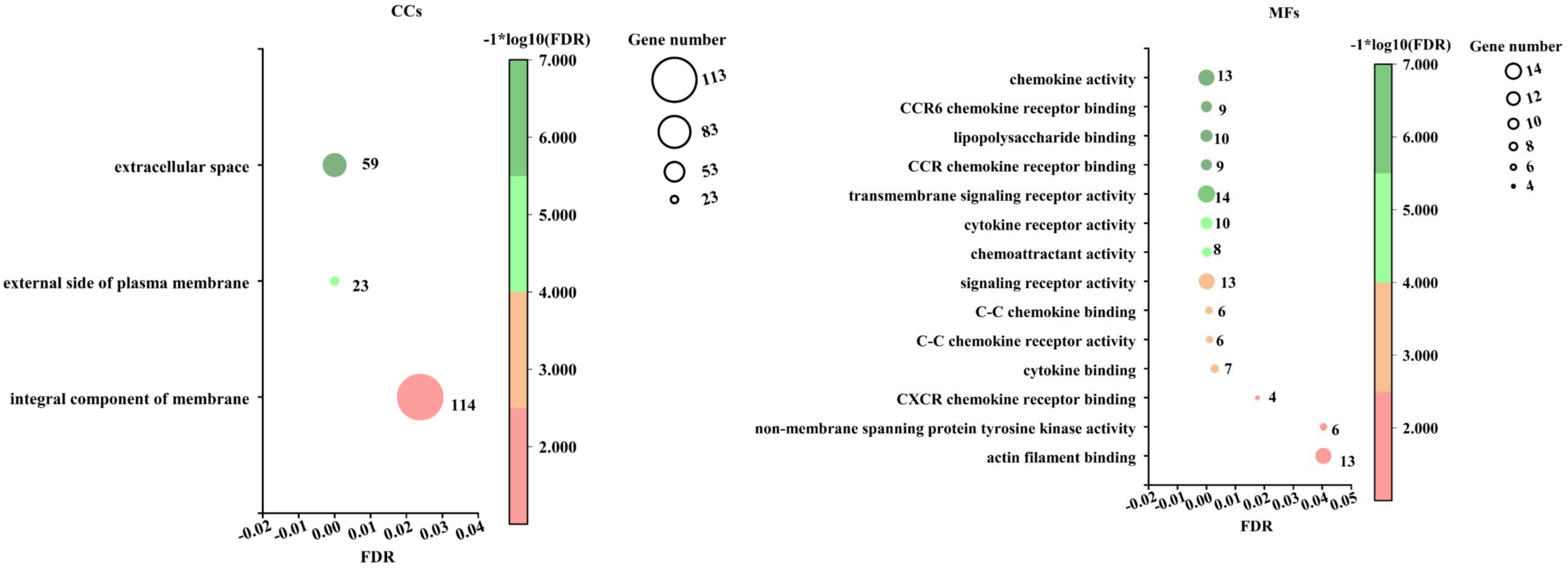

Figure S2. The most significant enriched CCs and MFs based on FDR in chicks

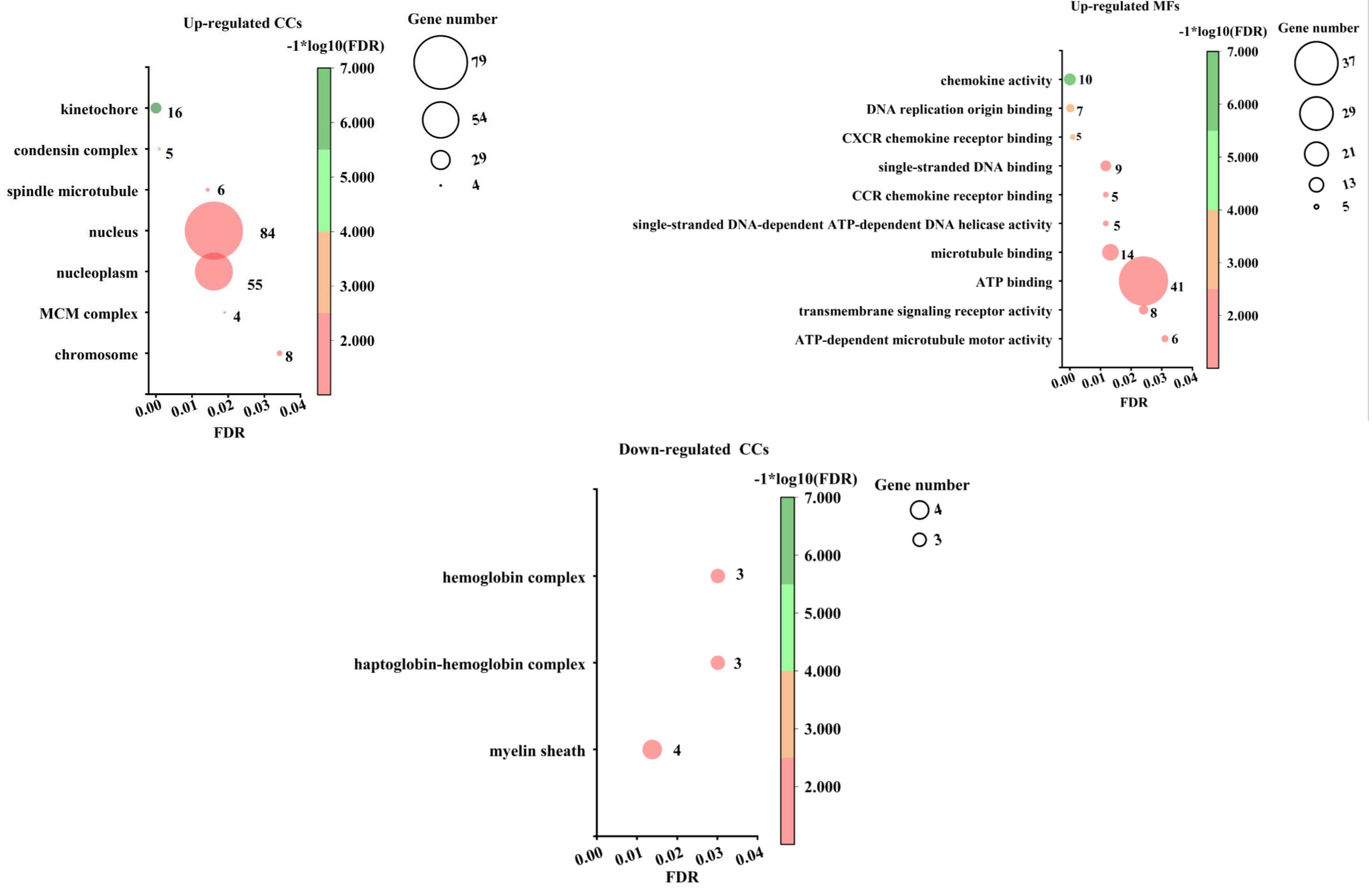

Supplement: Supplementary file 1 [file animals-13-01667-s001.zip › Supplementary materials Figures S1&S2.pdf]
