# Supplementary material for: Comparative Transcriptome Analysis Reveals the Innate Immune Response to Mycoplasma gallisepticum Infection in Chicken Embryos and Newly Hatched Chicks
Source: Animals (Basel). 2023 May 17;13(10):1667. doi: 10.3390/ani13101667 (PMC10215417; doi:10.3390/ani13101667)
Supplement: Supplementary file 1 [file animals-13-01667-s001.zip › Supplementary materials Tables S1-S4.pdf]

Table S1. Top 30 upregulated genes in the lung tissues of *MG*-infected chicken embryos

DEG	Log_2Fold change	Adjust-P value
IL8L2	8.973782	4.22E-10
IL4I1	8.292192	9.01E-24
AVD	8.112968	1.10E-229
CCLI7	7.942147	9.58E-07
LOC770026	7.871279	5.93E-07
P2RY4	7.472908	9.17E-05
ATP6V1G3	7.065767	0.00014
CCL26	6.859695	5.98E-13
CCL20	6.7652	0.001188
CCL4	6.712354	3.59E-09
PLEKHS1	6.693646	0.000418
CCLL4	6.485033	0.010681
CLEC5A	6.426947	1.13E-05
LOC112531168	6.426396	0.000258
XCR1	6.424492	0.007951
IL1R2	6.380867	4.75E-10
MSC	6.27775	0.003953
SAMD9L	6.246109	2.23E-09
PROK2	6.22687	8.84E-08
IL8L1	6.075514	5.63E-05
MLKL	6.022089	2.49E-10
LOC112533211	5.996647	0.000124
CD72L1	5.84905	7.68E-14
LOC101750289	5.818267	0.025238
LOC427029	5.802992	5.57E-19
OASL	5.66904	2.31E-14
TNFRSF6B	5.517577	8.78E-05
CXCL13L2	5.491891	1.62E-41
LOC417536	5.344942	0.000235
LOC101751902	5.343251	0.018943

Table S2. All 15 down-regulated genes in the lung tissues of *MG*-infected chicken embryos

DEG	Log_2Fold change	Adjust-P value
UNC93A	-6.55972	0.006338
AHSG	-5.3491	0.029024
ALPI	-5.09028	0.040388
C1QL2	-4.03883	0.014671
PPP4R4	-3.32034	0.038925
NCAN	-2.80507	0.007867
FAM179A	-2.7754	0.007394
NRN1L	-2.01916	0.022568
BDKRB2	-1.86191	0.033623
SLC6A2	-1.77201	5.26E-18
SMYD1	-1.70329	0.026774
TMC3	-1.63143	0.000218
PAK5	-1.59812	0.006353
ASIC4	-1.54147	0.041798
ACHE	-1.52729	0.009075

Table S3. Top 30 upregulated genes in the lung tissues of *MG*-infected newly hatched chicks

Gene name	Log_2Fold change	Adjust-P value
MMP7	12.04159862	0.000403
OLFM4	10.65407643	0.000322
LOC107051274	9.229779539	2.04E-11
SLC5A5	8.024048633	0.000135
CXCL13	7.46365811	4.76E-05
LOC112530909	6.851436226	0.002573
AICDA	6.839024468	6.83E-06
JCHAIN	6.599543805	9.83E-11
SPIC	6.561367942	0.000113
FFAR4	6.480470898	0.011065
GPR55	6.273344439	5.31E-05
VPREB3	6.176063932	0.014851
LOC770026	5.628905901	9.02E-05
NEUROD6	5.581582592	0.012157
CDC20B	5.496247892	0.018955
CCR10	5.239873323	0.000867
CFAP44	5.205267418	0.00396
CXCL13L2	5.034945813	0.000766
AKAP5	5.011569532	0.030183
CXCL13L3	4.920649007	0.024743
ELF5	4.872847995	0.046156
TNFRSF13B	4.840671894	9.63E-05
IL8L1	4.837174964	0.01823
DCSTAMP	4.602756438	3.74E-07
MOV10L1	4.524659113	0.002372
IL4I1	4.342169539	0.001511
TSPAN8	4.301919902	0.04184
CD72	4.27258542	4.67E-07
SPAG17	4.270448922	0.017749
CCL19	4.229992336	3.29E-07

Table S4. Top 30 downregulated genes in the lung tissues of *MG*-infected newly hatched chicks

Gene name	Log_2Fold change	Adjust-P value
ACSM4	-7.3408	0.01626855
DUOX1	-7.03437	0.004384207
LOC107054425	-6.9764	0.020557382
TYRP1	-6.10877	0.022226087
CNTN2	-6.0549	0.012513864
LOC101751348	-5.35551	0.041068471
ANKS4B	-5.24498	0.032168101
LOC771456	-5.16525	0.027635347
SSTR4	-4.90221	0.034948235
PLP1	-4.78708	0.004384207
HBE1	-4.26165	0.000346552
ASAH2	-4.21008	7.08E-05
HSPB9	-4.04651	0.003363128
NKX6-3	-4.00076	0.010844828
ADMP	-3.97935	0.009141828
HRH4	-3.95846	2.09E-07
PPP1R1C	-3.80963	0.020819931
PLLP	-3.7752	0.009385411
MUC1	-3.76874	3.81E-07
MIR451B	-3.71438	0.012861038
BHMT2	-3.60428	0.034948235
LOC112530278	-3.60073	0.01604615
GRM4	-3.58945	0.008229423
MIR144	-3.56593	0.003342736
TNNT2	-3.53145	0.018223144
CRYBB3	-3.36165	0.001332361
LOC112531995	-3.36107	0.029945154
RUNDC3A	-3.3408	0.003375379
NR4A3	-3.28842	6.95E-05
SKOR1	-3.19457	0.049832125
